# Posaconazole‐induced hypertension in children with cystic fibrosis

**DOI:** 10.1002/rcr2.822

**Published:** 2021-08-04

**Authors:** Rachael Marpole, Daniel K. Yeoh, Adelaide L. Withers

**Affiliations:** ^1^ Department of Respiratory and Sleep Medicine Perth Children's Hospital Perth Western Australia Australia; ^2^ Department of Infectious Diseases Perth Children's Hospital Perth Western Australia Australia; ^3^ Department of Oncology University of Melbourne, Sir Peter MacCallum Parkville Victoria Australia; ^4^ Wal‐Yan Respiratory Research Centre Telethon Kids Institute Perth Western Australia Australia

**Keywords:** allergic bronchopulmonary aspergillosis, cystic fibrosis, hypertension, posaconazole

## Abstract

Posaconazole is a triazole antifungal with a broad spectrum of activity against moulds including Aspergillus spp. Emerging data suggest posaconazole may be effective in the treatment of allergic bronchopulmonary aspergillosis (ABPA) complicating cystic fibrosis (CF). Rarely, posaconazole can cause pseudohyperaldosteronism, manifesting as hypertension and electrolyte abnormalities, with a number of cases recently reported in individuals without CF. We describe two cases of children with CF who developed hypertension, likely due to pseudohyperaldosteronism, following the initiation of posaconazole for the treatment of ABPA.

## Abstract 2

Posaconazole is a triazole antifungal with a broad spectrum of activity against moulds including Aspergillus spp. Rarely, it can cause pseudohyperaldosteronism. We describe two cases of children with cystic fibrosis who developed hypertension, likely due to pseudohyperaldosteronism, following the initiation of posaconazole for the treatment of allergic bronchopulmonary aspergillosis.

## INTRODUCTION

Posaconazole is a triazole antifungal with a broad spectrum of activity against moulds including Aspergillus spp. Emerging data suggest posaconazole may be effective in the treatment of allergic bronchopulmonary aspergillosis (ABPA) complicating cystic fibrosis (CF).^1^ Rarely, posaconazole can cause pseudohyperaldosteronism, manifesting as hypertension and electrolyte abnormalities, with a number of cases recently reported in immunocompromised patients who were not on steroids.^2^ We describe two cases of children with CF who developed hypertension, likely due to pseudohyperaldosteronism, following the initiation of posaconazole while on systemic steroids for the treatment of ABPA.

## CASE REPORT

### Case 1

A 9‐year‐old male (homozygous p.Phe508del) known to be colonized with *Pseudomonas aeruginosa* who had been taking ivacaftor/lumacaftor for 1 year developed cough and wheeze. His blood pressure (BP) was 100/60 mmHg. *Aspergillus fumigatus* complex was cultured from sputum and chest x‐ray showed coarsened bronchovascular markings with focal opacification. Clinical response to anti‐pseudomonal antibiotics and a short course of prednisolone were poor and IgE was elevated (1029 kU/L [<72 kU/L]). Itraconazole was initiated and oral prednisolone re‐commenced. Computed tomography (CT) imaging of the chest showed changes consistent with ABPA including new bronchiectasis and endobronchial secretions in all lobes with associated branching airspace opacity. Monthly pulsed intravenous (IV) methylprednisolone (15 mg/kg for 3 days) was commenced and prednisolone ceased. BP became elevated following methylprednisolone (maximum BP 125/90 mm/Hg) above the 95th centile for age and height (118/78 mmHg).

As itraconazole levels remained subtherapeutic despite dose adjustment, antifungal therapy was changed to posaconazole (delayed‐release tablets). Therapeutic levels (3.78 mg/L) were attained within 2 weeks. Prior to the second methylprednisolone pulse, BP was elevated (115/80 mmHg) and IgE had normalized (367 kU/L).

He remained clinically stable and, prior to the fifth methylprednisolone pulse, BP was elevated at 120/80 mmHg and increased to 165/110 mmHg following the pulse; amlodipine (5 mg) was commenced for hypertension. Hypokalaemia (3.0 [3.3–4.9 mmol/L]) and hypophosphataemia (0.79 [0.90–2.00 mmol/L]) were noted. Pulsed methylprednisolone was ceased following clinical improvement. Amlodipine was ceased 1 month later; however, BP remained elevated (130/75 mmHg) requiring amlodipine to be restarted. His IgE has remained low (85 kU/L) and symptoms of ABPA have not flared. Posaconazole was ceased after a total of 9 months; BP remained stable (on amlodipine). Amlodipine was ceased 4 months later when BP was normal.

### Case 2

A 7‐year‐old girl with CF (homozygous p.Phe508del) and episodic ABPA over the preceding 4 years developed worsening cough and deterioration in appearance of CT chest. Ivacaftor/lumcaftor had been recently commenced. Previous ABPA treatment included oral prednisolone, IV methylprednisolone, itraconazole, posaconazole and inhaled liposomal amphotericin. Posaconazole had previously been discontinued after a week due to elevation in hepatic enzymes and clinical response with itraconazole was poor, with associated difficulties attaining therapeutic levels. Use of oral steroids had been previously limited due to significant associated behavioural changes. Omalizumab was precluded due to severe needle phobia. She had previous episodes of hypertension with concurrent administration of ivacaftor/lumacaftor, itraconazole and steroids; however, these episodes were asymptomatic and self‐resolving.

Despite recent treatment with inhaled liposomal amphotericin and oral prednisolone, no clinical improvement was observed and serum IgE increased to 1223 kU/L. Baseline BP was 100/60 mmHg. Posaconazole 200 mg three times a day was initiated, with the plan to initiate mepolizumab as a steroid‐sparing agent. Two weeks following posaconazole initiation, she developed headache and facial flushing. BP was elevated to 136/98 mmHg. Posaconazole serum levels were high (8 mg/L), further doses were withheld and clonidine was started for hypertension. Mild hypokalaemia was present (3.0 mmol/L). Repeat CT imaging demonstrated new high attenuation endobronchial material in all lobes, consistent with worsening disease. Pulse methylprednisolone (22 mg/kg for 3 days) was given. Posaconazole was restarted at 200 mg twice daily and mepolizumab (40 mg monthly) commenced. On discharge, BP was controlled on clonidine between 96/40 and 110/75 mmHg.

Headaches and hypertension worsened despite clonidine and a reduced serum posaconazole level (4 mg/L). She redeveloped hypokalaemia (2.5 mmol/L). Posaconazole dose was reduced further (200 mg in the morning and 100 mg in the evening). Her BP control was maintained with clonidine and amlodipine (5 mg twice daily) at 105/60 mmHg and hypokalaemia resolved. Her IgE has reduced to 250 kU/L after 2 months of mepolizumab. Posaconazole was ceased after 4 months, her BP remains stable (105/60 mmHg) while antihypertensives are being weaned.

## DISCUSSION

We report two cases of hypertension with associated hypokalaemia following the initiation of posaconazole for the treatment of ABPA. The likely mechanism for hypertension in both these cases is posaconazole‐induced pseudohyperaldosteronism.

ABPA is a type 2 hypersensitivity reaction, mainly triggered by *A*. *fumigatus*.^3^ The aims of treatment are to downregulate the host's inflammatory response and to decrease the fungal burden. Steroids are the recommended first‐line therapy, with addition of triazole antifungals for refractory cases. The most commonly used antifungal is itraconazole, although some data support the use of posaconazole, including in children with CF.^1^ Emerging data suggest a potential role for biological agents including omalizumab and mepolizumab as steroid‐sparing agents.^3^

Ivacaftor/lumacaftor is a CF transmembrane conductance regulator modulator that is currently licenced in Australia for individuals with CF who are homozygous for the p.Phe508del mutation from 24 months of age.^4^ Hypertension has been reported as an uncommon side effect.

In terms of hypertension as a side effect from oral antifungals, both posaconazole and itraconazole can cause hypertension, and voriconazole does not cause hypertension.^4^ Azole‐induced pseudohyperaldosteronism is mediated by inhibition of adrenal steroidogenic enzyme CYP11B1.^5^ This inhibition results in accumulation of excess mineralocorticoids (11‐deoxycorticosterone and 11‐deoxycortisol) leading to hypertension and hypokalaemia. Cortisol insufficiency may also occur, similar to the presentation of genetic forms of 11 beta‐hydroxylase deficiency.^5^

Unfortunately, in both cases described here, the potential pseudohyperaldosteronism was not recognized until the resolution of hypertension and confirmatory urine and blood tests to assess the renin–angiotensin–aldosterone pathway were not obtained. The likely mechanism in both cases was posaconazole‐induced pseudohyperaldosteronism as persistent BP elevation was only noted after the initiation of posaconazole. Concomitant administration of ivacaftor/lumacaftor and posaconazole may reduce posaconazole levels; however, effects on BP are unclear.^4^ Systemic steroid administration is a possible confounder in this case, as steroids may cause hypertension and hypokalaemia.^4^

Close monitoring of BP, serum electrolytes and trough posaconazole levels should be implemented following the initiation of posaconazole in patients with ABPA to ensure early detection of this adverse effect.^5^ Spironolactone has been used to ameliorate the effect of pseudohyperaldosteronism. Alternatively, other triazoles including voriconazole do not inhibit CYP11B1 and may be considered if ongoing antifungal therapy is indicated.

In conclusion, we describe two cases of likely posaconazole‐induced pseudohypoaldosteronism presenting with hypertension and hypokalaemia in children receiving treatment for ABPA complicating CF. Hypertension may be multifactorial in this setting, given the frequent concomitant administration of steroids, ivacaftor/lumacaftor together with posaconazole. When prescribing posaconazole in children with ABPA, respiratory clinicians should be aware of this potential adverse effect and ensure appropriate monitoring is in place.

## FUNDING INFORMATION

Funding was received from the Child and Adolescent Health Service Research Fellowship.

## CONFLICT OF INTEREST

None declared.

## ETHICS STATEMENT

Appropriate written informed consent was obtained for publication of this case report.

## AUTHOR CONTRIBUTIONS

Rachael Marpole prepared the first draft of this paper under the supervision of Adelaide L. Withers. The draft was revised by Daniel K. Yeoh.

## References

[1] Patel D , Popple S , Claydon A , Modha DE , Gaillard EA . Posaconazole therapy in children with cystic fibrosis and Aspergillus‐related lung disease. Med Mycol [Internet]. 2020;58(1):11–21. Available from: https://academic.oup.com/mmy/article/58/1/11/5381954 30877757 10.1093/mmy/myz015

[2] Nguyen MVH , Davis MR , Wittenberg R , McHardy I , Baddley JW , Young BY , et al. Posaconazole serum drug levels associated with pseudohyperaldosteronism. Clin Infect Dis [Internet]. 2020;70(12):2593–8. Available from: https://academic.oup.com/cid/article/70/12/2593/5546442 31403165 10.1093/cid/ciz741 PMC7286374

[3] Sunman B , Ademhan Tural D , Ozsezen B , Emiralioglu N , Yalcin E , Özçelik U . Current approach in the diagnosis and management of allergic bronchopulmonary aspergillosis in children with cystic fibrosis. Front Pediatr [Internet]. 2020;8(October):582964. Available from: https://doi-org.pklibresources.health.wa.gov.au/10.3389/fped.2020.582964 33194914 10.3389/fped.2020.582964 PMC7606581

[4] MIMS . Australia online database [Internet]. MIMS Australia. 2020 [cited 2021 Apr 9]. Available from: http://www.mimsonline.com.au.

[5] Boughton C , Taylor D , Ghataore L , Taylor N , Whitelaw BC . Mineralocorticoid hypertension and hypokalaemia induced by posaconazole. Endocrinol Diabetes Metab Case Rep [Internet]. 2018;2018(February):1–5. 10.1530/EDM-17-0157 PMC5813713 29472988

